# Improving PFSA Membranes Using Sulfonated Nanodiamonds

**DOI:** 10.3390/membranes13080712

**Published:** 2023-08-01

**Authors:** Alexandr V. Shvidchenko, Alexei S. Odinokov, Oleg N. Primachenko, Iosif V. Gofman, Natalia P. Yevlampieva, Elena A. Marinenko, Vasily T. Lebedev, Alexander I. Kuklin, Yuri V. Kulvelis

**Affiliations:** 1 Ioffe Institute, 194021 St. Petersburg, Russia; 2 Russian Research Center of Applied Chemistry, 193232 St. Petersburg, Russia; oas9@mail.ru; 3 Institute of Macromolecular Compounds, Russian Academy of Sciences, 199004 St. Petersburg, Russia; alex-prima@mail.ru (O.N.P.); gofman@imc.macro.ru (I.V.G.); emarinenkospb@gmail.com (E.A.M.); 4 Physical Faculty, St. Petersburg State University, 198504 St. Petersburg, Russia; n.yevlampieva@spbu.ru; 5 Petersburg Nuclear Physics Institute Named by B.P. Konstantinov of National Research Center “Kurchatov Institute”, 188300 Gatchina, Russia; lebedev_vt@pnpi.nrcki.ru; 6 Frank Laboratory of Neutron Physics, Joint Institute for Nuclear Research, 141980 Dubna, Russia; alexander.iw.kuklin@gmail.com

**Keywords:** nanodiamonds, Aquivion, perfluorosulfonic acid membrane, proton exchange membrane, proton conductivity, small-angle neutron scattering, sulfonating

## Abstract

Aquivion^®^-type perfluorosulfonic acid membranes with a polytetrafluoroethylene backbone and short side chains with sulfonic acid groups at the ends have great prospects for operating in hydrogen fuel cells. To improve the conducting properties of membranes, various types of nanofillers can be used. We prepared compositional Aquivion^®^-type membranes with embedded detonation nanodiamond particles. Nanodiamonds were chemically modified with sulfonic acid groups to increase the entire amount of ionogenic groups involved in the proton conductivity mechanism in compositional membranes. We demonstrated the rise of proton conductivity at 0.5–2 wt.% of sulfonated nanodiamonds in membranes, which was accompanied by good mechanical properties. The basic structural elements, conducting channels in membranes, were not destroyed in the presence of nanodiamonds, as follows from small-angle neutron scattering data. The prepared compositional membranes can be used in hydrogen fuel cells to achieve improved performance.

## 1. Introduction

The development of the hydrogen energy industry, based mainly on membrane technologies, is one of the most important fields in the decarbonization of economics. The fuel cell (FC) industry is major technology to implement hydrogen energy into practical use and shows a strong trend of increasing in future decades [1,2,3,4]. In addition to hydrogen energy, the implementation of renewable energy sources (solar, wind, hydropower) should be considered in trends of decarbonization [5]. Most renewable energy sources are intermittent, not continuously available for consumers [5], and thus may serve generally as additional sources of energy, which require the presence of a stable primary source. Normally, the renewable energy can be used for stationary power devices, while transport vehicles need special vehicle-to-grid technology to replace the gasoline-powered engine and the use of renewable energy sources [6]. To solve these problems, special energy storage systems for power grids should be developed, where PEM (proton exchange membranes or polymer electrolyte membranes) may be involved, due to their proved effectivity in electrochemical energy conversion systems [5,7]. Thus, renewable energy sources and hydrogen energy can be effectively combined for stable energy generation [8].

PEM fuel cells are based mainly on perfluorosulfonic acid (PFSA) Nafion^®^-type membranes, which were first implemented into working power devices.

Nafion^®^ membranes, based on a polytetrafluoroethylene backbone with sulfonic acid groups at the end of the long side chains, demonstrated high physical and mechanical properties, chemical resistance, and a high ionic (proton) conductivity [3,9,10,11,12,13,14,15]. Later research demonstrated that PFSA Aquivion^®^ membranes with shortened side chains have advantages over Nafion^®^ due to larger values of proton conductivity and better thermal stability [16,17,18,19]. Further improving the membranes’ properties may involve various types of modifying additives embedded into the membranes’ structure [9,20,21,22,23,24,25,26,27,28,29].

One of the possible modifiers for PFSA membranes can serve diamond nanoparticles (nanodiamonds). Nunn et al. reports various classes of nanodiamond particles, methods of their synthesis, and structural features [30]. Size, shape, crystallographic core, surface chemical composition, internal defects, impurities, and presence of sp^2^ carbon in nanodiamonds are observed, as well as their relationship with various fields of application [30]. Nanodiamonds and nanostructures based on diamonds have remarkable electrochemical properties and may be used in the fields of electroanalysis, energy storage, as nanoelectrodes, etc. [31]. Diamond nanoparticles, produced by detonation synthesis (detonation nanodiamonds, DND) are of special interest. DND particles with a typical mean size of 4–5 nm have a chemical inert core and may have various functional groups on the surface, allowing them to regulate the sign of ζ-potential—positively or negatively charged surfaces in aqueous or other mediums. Postnov et al. reported composites with nanodiamonds based on Nafion^®^ and Aquivion^®^ materials, where a rise of proton conductivity was demonstrated under low humidity conditions [32].

Recently, we have studied PFSA Aquivion^®^-type compositional membranes with either negatively charged carboxylated nanodiamonds [33] with a negative ζ-potential (DND Z−) [34,35,36] or positively charged protonated nanodiamonds [37] with a positive ζ-potential (DND Z+) [36,38]. We demonstrated that DND Z+ distributed in the membrane matrix much more homogeneously, forming rough aggregates below 300 nm in size, while DND Z− strongly agglomerated to micron-size particles. The homogeneous distribution of DND Z+, due to their attraction to sulfonic acid groups of the polymer, resulted in a more effective rise of proton conductivity in compositional membranes, compared to DND Z−, while saving their mechanical properties. DND Z+ at 0.5 wt.% embedded in compositional membranes have significantly improved membrane properties and performance in membrane-electrode assemblies (MEA) at temperatures up to 120 °C [38].

Sulfonated compounds embedded as fillers for PFSA composites are of greatest interest in order to increase the number of ionogenic sulfonic acid groups in the membrane involved in the proton exchange mechanism [39,40,41,42]. Sulfonated nanodiamonds, if they have been prepared, are excellent candidates for this purpose. Kuznetsov et al. described a two-stage synthesis of sulfonated DNDs [43], when nanodiamonds, functionalized with phenyl radicals, were sulfonated in oleum, resulting in a -SO_3_H-group attached to the diamond particle through a benzene ring. Lei et al. reported a one-stage synthesis of sulfonated DNDs based on their surface modification with sulfanilic acid [44]. Again, the sulfonic acid group in this case is chemically connected with the DND surface through an additional benzene ring.

To further improve the distribution of DND in membranes and their functional properties, in the current work, we applied a direct sulfonation of DND Z+ particles by oleum and introduced sulfonated DNDs into Aquivion^®^-type membranes for the first time.

## 2. Experimental Procedure

### 2.1. Sample Preparation

#### 2.1.1. Sulfonation of Nanodiamonds

Sulfonation was applied using oleum on the initial protonated DND with a positive ζ-potential (DND Z+), obtained from industrial nanodiamonds produced by the Scientific and Production Closed Joint Stock Company “Sinta” (Minsk, Belarus), using the approach described by Williams et al. [37]). The industrial DND was annealed in molecular hydrogen at 600 °C for 3 h. A hydrosol of annealed DND was prepared by ultrasonic dispersion of DND powder in deionized water. The prepared hydrosol was centrifuged (1.8 × 10^4^× *g*, 40 min) to separate remaining aggregates from individual 4–5 nm diamond particles. The particles of DND Z+ had a mean size of 4–5 nm and ζ-potential +70 mV (Hückel) in the supernatant. The nature of the protonated DNDs still remains discussable [45,46].

Sulfonation of the DND Z+ sample was carried out as follows. A glass tube of 10 mL volume was filled with 0.63 g HgSO_4_, 1.13 g DND Z+, and 6.55 g of oleum (SO_3_ 65% in H_2_SO_4_). The tube was heated in a sealed steel ampoule (pressure under 15 MPa) at 150 °C for 4 h, then cooled to the ambient temperature for 3 h. The obtained black reaction mass was diluted by the slow addition of 10 mL of distilled water and mixing. The obtained mixture was washed out by adding 30 mL of deionized water, ultrasonication (20 min), centrifugation (1.8 × 10^4^× *g*, 30 min), and decantation multiple times. The resulting sediment was dried to a constant weight (237 mg) and mixed with 12.28 g of dimethylformamide (DMF). The mixture was treated in ultrasonic baths (60 kHz, 20 min and 22 kHz, 20 min) and centrifuged (1.8 × 10^4^× *g*, 5 min.) to isolate a supernatant with small particles (DND-S sample) from aggregates because sulfonated diamonds are partially dispersed in DMF. The supernatant contained 47% (110 mg) from an initial amount of DND. The resulting sol of DND-S 0.5 wt.% in DMF was stable for a prolonged period, having a mean particle size of 8–10 nm and a positive ζ-potential: 21 mV (Smoluchowski) or 31 mV (Hückel). This is consistent with the results described earlier for aqueous suspensions of DND Z+ [47], demonstrating a weak association of DND-S particles in DMF, which is expected when a less polar medium is used.

#### 2.1.2. Preparation of Compositional Membranes

A perfluorinated copolymer with short side chains, having an equivalent weight (EW) of 890 g-eq/mol, was synthesized by an aqueous-emulsion technique [48]. A monomer FS-81 (perfluoro-3-octapentensulfonilfluoride) was copolymerized with tetrafluoroethylene (TFE) [16,48]. Membranes were prepared by a solution casting technique using a DMF solution of a –SO_3_Li form of copolymer (2 wt.%), when the solvent was removed at 70–80 °C followed by annealing at 170–200 °C for 1–2 h and reducing to a –SO_3_H form, according to the adopted method [18]. This technique provides high thermal stability of the obtained membranes, as demon-strated in Figure S1. Compositional membranes were prepared from the mixture of suspensions of DND-S in DMF and perfluorinated copolymer in DMF. The DND-S suspension in DMF was prepared from dried DND-S hydrosol after removing water and dispersing in DMF using an ultrasonic generator. Then, 2 wt.% of copolymer solution was mixed with 0.5 wt.% DND-S suspension with the variable ratio of the components to obtain an assigned DND content in the membranes from 0.25 to 5 wt.% after evaporating the solvent. The adopted method of membrane preparation for pure polymers without diamonds was repeated in the presence of DND-S to obtain compositional membranes. Thus, six membranes were prepared with 0, 0.25, 0.5, 1, 2, and 5 wt.% of DND-S. The thickness of the membranes in air-dry conditions was 60–70 μm.

### 2.2. Methods of Investigation

#### 2.2.1. Energy-Dispersive X-ray Analysis

Elemental analysis was carried out using a scanning electron microscope (Tescan Vega 3) equipped with an energy-dispersive X-ray (EDX) analysis unit (Oxford Instruments). The accelerating voltage of the electron beam was 30 kV (penetration depth is ~1 μm). The electron beam was directed normally to the silicon substrate surface with a diamond film. The pressure in the chamber was ~10^−7^ mm Hg.

#### 2.2.2. Fourier-Transform Infrared (FTIR) Spectroscopy

FTIR spectra of the initial and sulfonated DNDs were recorded using an IR-Fourier-spectrometer InfraLum FT-08 (Lumex, St. Petersburg, Russia) with diffuse reflection accessory PIKE EasyDiff. Sample powders were placed in the sample cups and were heated in air at 80 °C for 1 h to reduce the interference from the adsorbed water. Heated cups were transferred into the sample chamber of the spectrometer to measure the FTIR spectra.

#### 2.2.3. ζ-Metric Titrations

Electrophoretic mobilities of the DND particles in hydrosols were analyzed by the Phase Analysis Light Scattering using Zetasizer Nano ZS (Malvern Instruments, Malvern, UK). Values of ζ-potential were calculated from electrophoretic mobilities using Henry’s equation [49]. HCl and NaOH aqueous solutions with concentrations of 0.1 M each were used as acid and base titrants. pH values of hydrosols were measured using an ion meter I-160 (Gomel Plant of Measuring Instruments, Gomel, Belarus) equipped with an H^+^-sensitive glass electrode and a silver chloride reference electrode.

#### 2.2.4. Proton Conductivity Measurements

The impedance spectroscopy method was used to measure the proton conductivity at 20 and 50 °C in an equilibrium state of saturation with water. Membranes were conditioned in boiling water (100 °C) for 1 h as an equivalent of ultimate moisture content (RH = 100%). A Z-3000X impedance meter (Elins, Moscow, Russia), using a measuring cell with stainless steel electrodes in a four-electrode scheme, was used in a frequency range of 10 Hz–150 kHz. Equation (1) was used to calculate the proton conductivity (σ_H_):

σ_H_ = L/(R × h × b). (1)

σ_H_ is the membrane’s specific conductivity of the (S·cm^−1^), L is the distance between voltage electrodes (cm), and R is the measured protonic resistance (Ohm) of the membrane with an average thickness h (cm) and an average width b (cm).

#### 2.2.5. Stress-Strain Mechanical Tests

Mechanical properties of the membranes were tested at an AG-100X Plus universal setup (Shimadzu Corp., Kyoto, Japan) at the stretching velocity of 100 mm·min^−1^, using the uniaxial extension mode. The working length of the membrane samples was 25 mm. Controlled climatic conditions were applied during the tests: relative humidity (RH) in the air of 50 ± 2% and a temperature of 23 ± 1 °C. Elastic modulus E, yield strength σ_Y_, ultimate tensile strength σ_T_, and ultimate deformation before destruction ε_D_ were defined.

#### 2.2.6. Small-Angle Neutron Scattering

Small-angle neutron scattering (SANS) was performed at the YuMO instrument (Joint Institute for Nuclear Research, Dubna, Russia) in the range (dynamic range also [50]) of momentum transfer q = (4π/λ) sin(θ/2) = 0.07–6 nm^−1^, where θ is the scattering angle, and the neutron wavelength is λ = 0.5–8 Å [51]. This q-range allows for revealing fine structure peculiarities on a scale of ~2π/q ~1–100 nm. Neutron scattering curves (scattering intensities, I vs. momentum transfer, q) were recalculated to thicknesses of the samples and normalized to absolute values of the scattering cross-section dΣ/DΩ (q) using vanadium as a standard for absolute intensity calibration in the SAS program package [52].

To obtain optimal scattering intensity, samples were packed in several-layer stacks for measurements. All samples were studied in an air-dry condition and measured at ambient temperature, 20 °C.

#### 2.2.7. Scanning Electron Microscopy

The surface structure of the prepared membranes was studied using scanning electron microscopy (SEM) at Zeiss AURIGA Laser (Carl Zeiss, Jena, Germany) multifunctional analytical system with crossed ion and electron beams. The device, equipped with a GEMINI^®^ electron optical column (Carl Zeiss, Jena, Germany), used a field emission cathode as an electron source. SEM images were obtained at secondary electron detectors (In-Lens and an Everhart-Thornley SE2 (Carl Zeiss, Jena, Germany)). The magnification parameter at an accelerating voltage of 15 kV in the SEM mode was 12× −1,000,000×; the spatial resolution was 1 nm. The working accelerating voltage was in the range of 0.1–30 kV, and a beam current was 10 pA–20 nA. The SmartSEM^®^ 6.00 software package (Carl Zeiss, Jena, Germany) was used to process the resulting images.

## 3. Results and Discussions

### 3.1. Identification of Sulfonated Nanodiamonds

The EDX method was used to study the DND-S powder and initial protonated DND Z+ to discover the results of sulfonating. It was found that the amount of S rises from 0.07 to 0.20 at.% after sulfonating, which means that a number of S atoms rises from 6 to 16–17 per one DND particle (size 4–5 nm, 8000 C atoms).

The FTIR spectra of DND Z+ and DND-S were recorded to determine if S atoms form –SO_3_H-groups on the surface.

**Figure 1:**
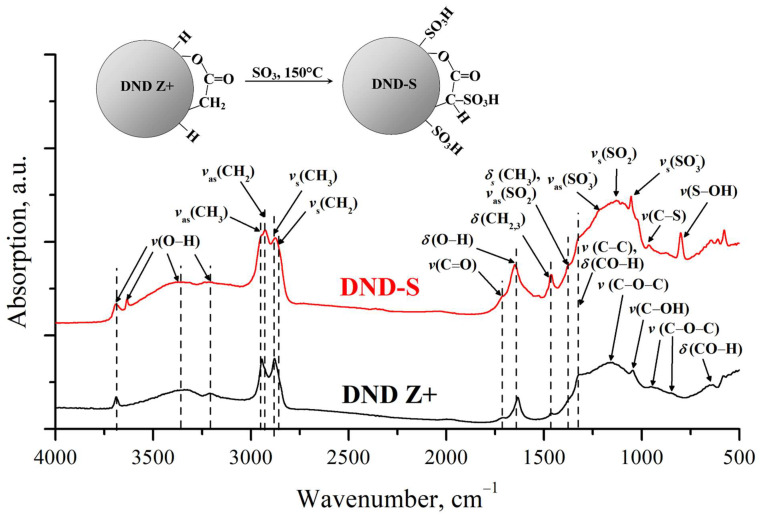
FTIR spectra of nanodiamonds before (DND Z+) and after sulfonation (DND-S).

Figure 1 shows the obtained FTIR spectra of the studied DNDs. Analysis of the characteristic bands of the DND Z+ surface functional groups shows that hydrocarbon groups CH_n_ are present on the particles’ surface. They correspond to a set of bands in the region 3000–2800 cm^−1^, related to symmetric and asymmetric stretching vibrations, as well as weak bands 1460 cm^−1^ and 1375 cm^−1^, corresponding to their bending vibrations. The spectrum also contains characteristic bands 3350, 3400, and 1635 cm^−1^ related to adsorbed water. In addition, the presence of free hydroxyls was revealed (3695 cm^−1^). A number of bands corresponding to ketones (1710 cm^−1^), ethers (1160 and 846 cm^−1^), and esters (1710, 1160, and 952 cm^−1^) were observed in the 1750–500 cm^−1^ spectral region. Bands corresponding to stretching and bending vibrations of hydroxyl groups in the configuration of secondary alcohols (1325, 1043 and 650 cm^−1^) also appeared in this region. The band at 1325 cm^−1^ can be also interpreted as C–C stretching vibration of the diamond lattice [53].

Sulfonation of DND Z+ leads to a change in the composition of its surface functional groups. A number of bands of the –SO_3_H-group stretching vibrations appear: SO_2_ (1380 and 1124 cm^−1^), the ionized groups –SO_3_^−^ (1220 and 1050 cm^−1^), C–S bonds (962 cm^−1^), and S–OH (798 cm^−1^) [54]. Our FTIR results, demonstrating the presence of sulfonic acid groups on the nanodiamond surface, agree well with work by Lei et al., who used sulfanilic acid to functionalize nanodiamonds with –OH groups [44].

**Figure 2:**
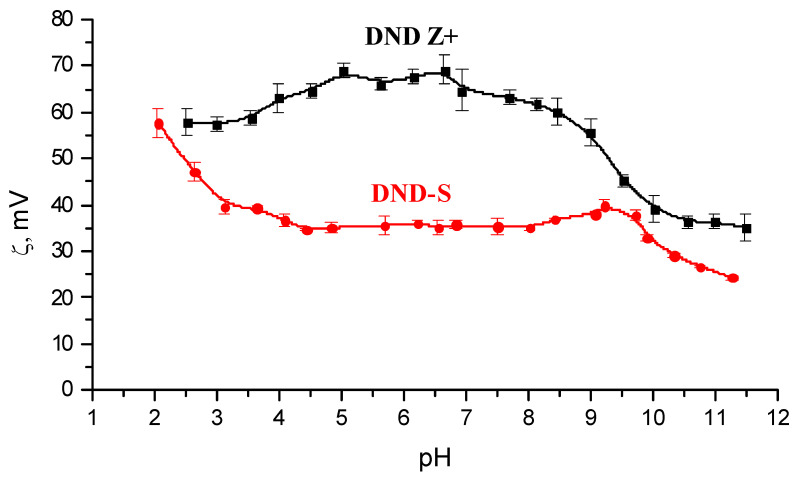
ζ-potentials of initial (DND Z+) and sulfonated (DND-S) nanodiamonds as a function of pH in an aqueous medium.

The results of ζ-metric titration also indicate the addition of sulfonic acid groups to the nanodiamond surface. The dissociation of these groups leads to a decrease in ζ-potential values of sulfonated particles compared to ζ-potentials of the initial particles in a wide range of pH (Figure 2). The observed ζ-potential values convergence of the studied samples in the strongly acidic pH region indicates that the association of sulfonic acid groups occurs; as a result, their influence on the positive charge of the particles disappears. The DND-S particles also turned out to be less stable than initial particles in the alkaline region. The stability region of sulfonated particles is limited to pH 9.5, while DND Z+ has a stability in the region up to pH 10.5.

### 3.2. Proton Conductivity of the Membranes

**Figure 3:**
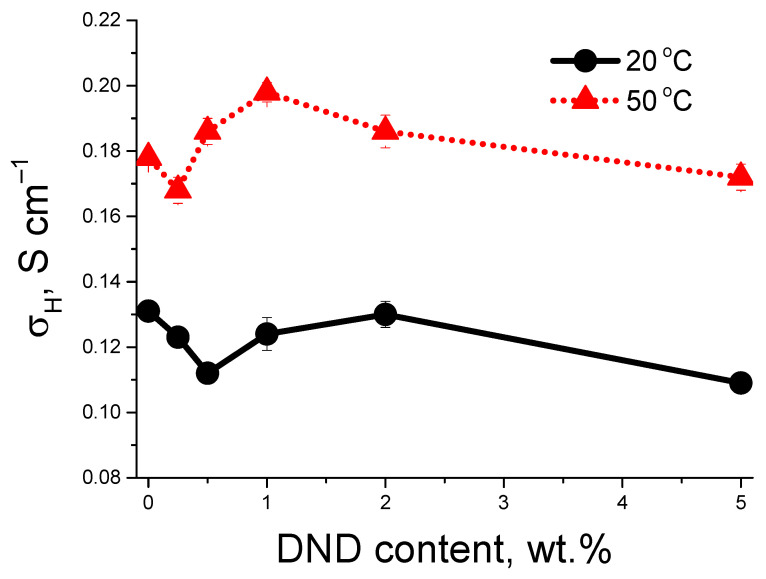
Proton conductivity of compositional membranes at 20 and 50 °C vs. DND content.

Figure 3 shows proton conductivity vs. DND content in membranes. At 20 °C, the conductivity in the compositional membranes decreases after DND-S loading to 0.5 wt.%, further filling results in the rise of conductivity with the local maximum at 2 wt.% of DND. At 50 °C, the doping of nanodiamonds results in the maximum of conductivity at 1 wt.% of DND, which is 10% higher than in the pure membrane without DND.

This complicated behavior of conductivity may be realized due to the nature of DND-S, having both positive and negative charges. At low DND content, DND particles, rarely located in the membrane, do not create a connected network as an additional pathway for protons, but may block some conducting channels, which leads to a decrease in conductivity. At higher DND-S contents, the connected network is created, which may help the protons to move along the membrane. The temperature should be an accelerating factor. The measured rise of conductivity at 50 °C allows for the expected larger effect at the typical working temperatures of these membranes ~130 °C.

We performed structural investigations using the SANS and SEM methods to find the peculiarities of DND-S distribution in membranes.

### 3.3. Mechanical Tests of the Membranes

**Figure 4:**
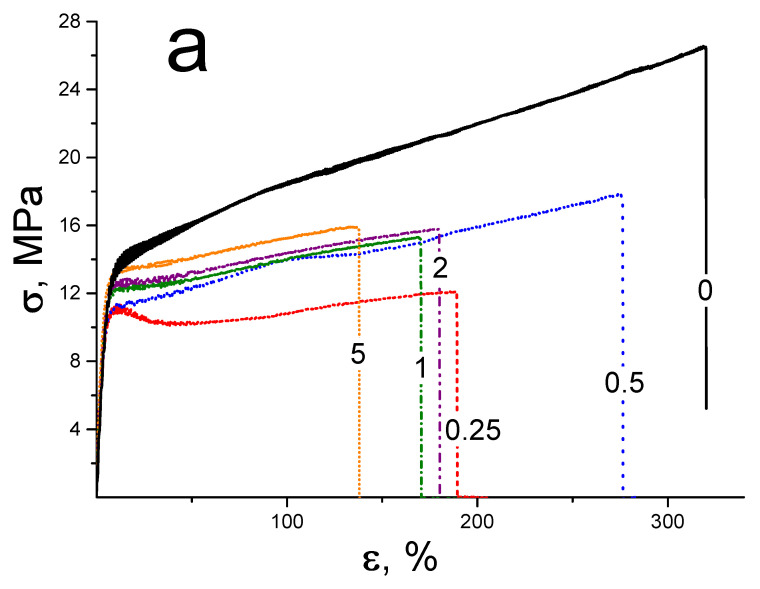

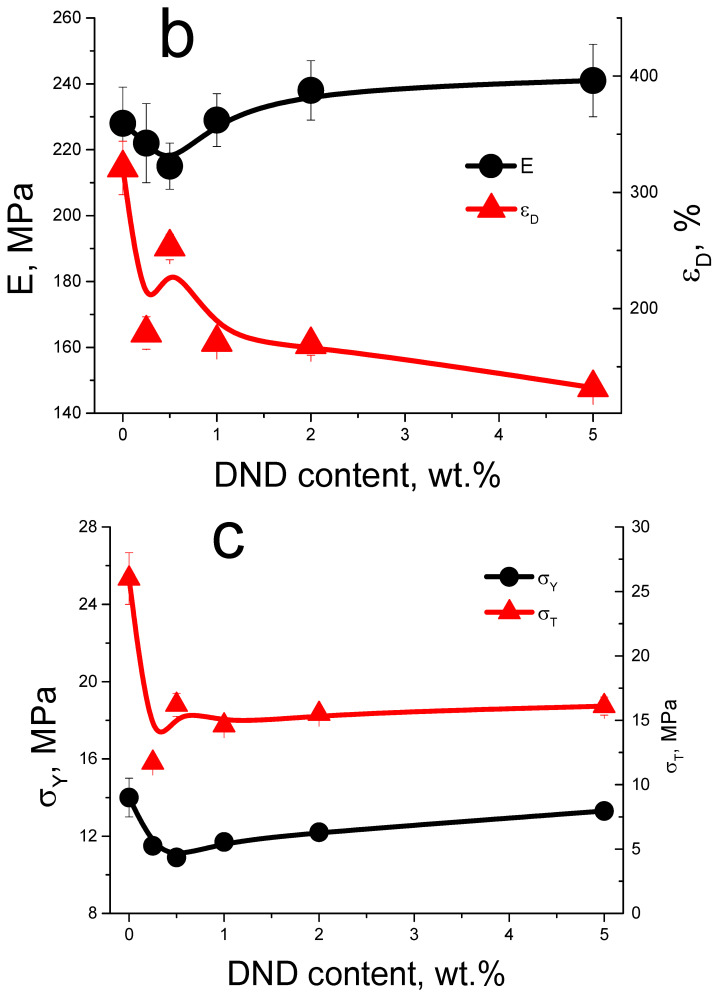
Mechanical properties of compositional membranes: (**a**) stress-strain profiles (wt. percentage of DND in membranes is displayed on the curves), (**b**) Young’s modulus E and ultimate deformation before destruction ε_D_, (**c**) yield strength σ_Y_ and ultimate tensile strength σ_T_.

Figure 4a demonstrates the measured stress-strain curves. All tested membranes are low-modulus polymeric materials (elastic modulus ~215–240 MPa) with high-strain resources (ultimate deformation before destruction for compositional membranes is of 130–250%). All tested membranes demonstrate a clearly identified transition over the yield strength (pronounced inflection on stress-strain curves) at stains of ~5–10%. This transition looks like the local maximum for the curve with a low DND-S content (0.25 wt.%). After this transition, the area of deformation strengthening of the material is observed.

While DND-S are introduced into the polymer matrix, a non-monotonic dependence of the mechanical properties was found (Figure 4b,c). At DND-S content ≤ 0.5 wt.%, rigidity of the films (elastic modulus and yield strength) decreases. A further increase in DND-S concentration in membranes leads to growth of these parameters and a decrease in ultimate deformation. The tendency of Young’s modulus to rise, as well as the decrease in ε_D_ and σ_T_, was also found earlier for similar membranes filled with protonated nanodiamonds [38], as well as for carboxylated nanodiamonds [34]. Parameter σ_Y_ in the current work and in the abovementioned cases is close to constant value with some deviations, demonstrating good elastic properties of membranes when DND has little effect on their elastic properties even at a high DND content.

### 3.4. Structural Studies of the Membranes by Small-Angle Neutron Scattering

**Figure 5:**
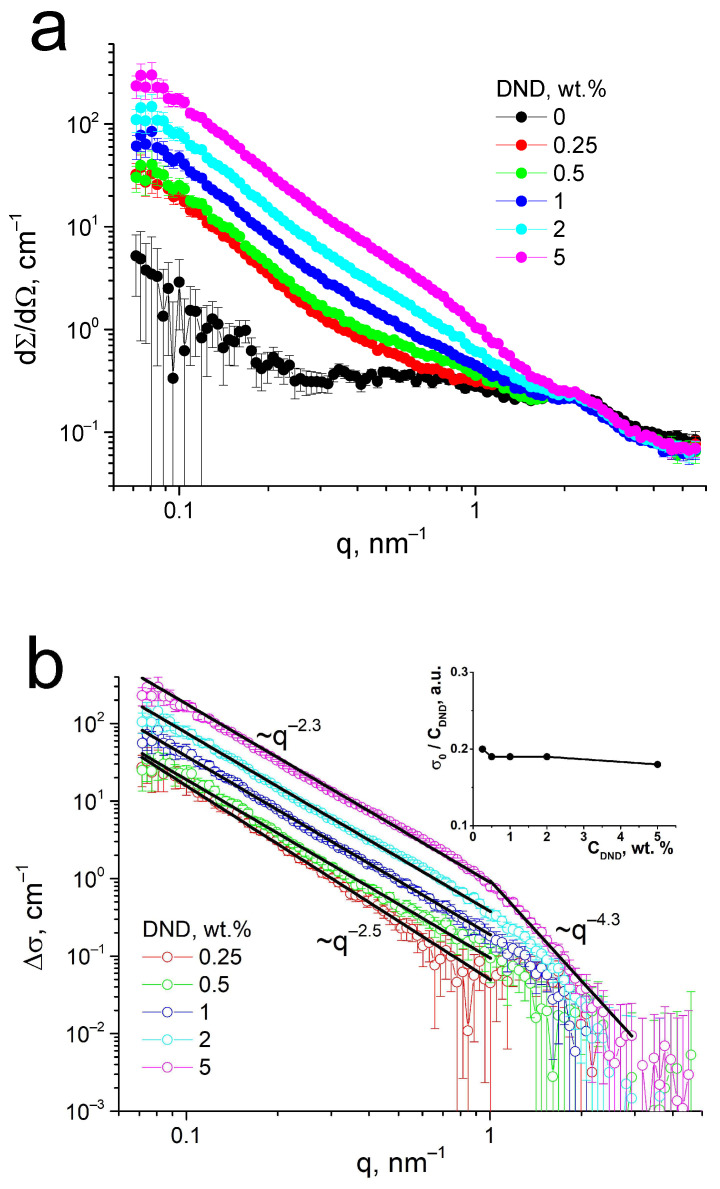
Small-angle neutron scattering (SANS) on dry Aquivion^®^-type membranes with sulfonated DNDs: (**a**) SANS profiles of membranes with a DND content of 0–5 wt.%; (**b**) differential SANS curves of membranes without DND subtracted from compositional membranes with DNDs, demonstrating the DND distribution in membranes: the points are experimental data, solid lines are power-law approximations, and the inset shows the intensity of the power-law fitting normalized by the DND content in membranes vs. the DND content.

SANS was performed to find the fine structure features of the compositional Aquivion^®^-type membranes with DND-S. Figure 5 shows SANS curves dΣ/dΩ(q), where the remaining of the ionomer peak is detected for all studied membranes. Similar profiles were earlier found for Aquivion^®^-type membranes with other types of DNDs—carboxylated DND with a negative charge [34] and protonated DND with a positive charge [38]. The ionomer peak remained almost unchanged in both series of the membranes, being a little more blurred at the presence of positive DNDs, while the distribution of positively charged DNDs in membranes was found to be much more uniform due to the coulombic attraction of DND to negatively charged sulfonic acid groups of the copolymer. Negatively charged carboxylated DNDs were not prone to attract either to sulfonic acid groups or to the backbone of the copolymer and formed a separate phase in the membranes on the border of hydrophilic and hydrophobic parts of the copolymer. In this work, sulfonated DNDs, being prepared from positively charged protonated DNDs, have obtained –SO_3_H-groups, negatively charged in the polar medium, but the total sign of ζ-potential of DND-S remained positive. Thus, the structure of such composites is of special interest. The remaining of the ionomer peak at Figure 5a demonstrates that the correlation between fine structural elements of the membranes—the conducting channels—is not disturbed, with a typical distance between neighboring channels R ~2π/q ~3 nm [17,55]. So, the presence of DND-S, as well as two other types of DNDs, does not change the general channel structure of the compositional membrane.

SANS at Figure 5a shows an additional wide peak (matrix knee) for pure Aquivion^®^-type membranes without DNDs in the q-range 0.4–1.0 nm^−1^, which disappears at the presence of DNDs. This peak demonstrates an ordering of channel bundles at a distance in the range of ~10–20 nm, which was described earlier [38] and is typical for Aquivion^®^ in dry conditions. When this peak disappears, the membrane structure at these scales may become more uniform at the presence of the DNDs. Similar behavior in compositional membranes was also observed for two other types of DNDs used earlier as fillers for PFSA membranes [34,38]. So, DND particles, which do not disturb conducting channels, may be localized in clusters on the next structural level—on a scale between bundles of channels. As in our previous work [38], we performed a subtraction of the SANS data for the pristine membrane from the curves of the compositional membranes to find the diamond contribution to scattering from polymer-diamond composites (Figure 5b). As a diamond scatters much more than a polymer at q < 1 nm^−1^, the analysis of the differential curves ∆σ(q) will give a good definition of how diamond particles are distributed in membranes even if the structure of the polymer in compositional membranes may undergo some changes. It was found that at low q < 1 nm^−1^, all differential SANS curves correspond to the power law ∆σ(q) = σ_0_·q^−D^ with a slope of D = 2.3–2.5. It corresponds to the scattering from fractal objects [56,57,58]—clusters of DND particles with a fractal dimension D. Diamond particles, associated into branched chain clusters in DND hydrosols [47,59,60,61], and dispersions in other solvents have a similar behavior. The area at q > 1 nm^−1^, demonstrating a power law with a slope of 4.3, corresponds to primary diamond particles with sharp facets [59]. Similar to the earlier-studied membranes with positively charged DNDs [38], the diamond component retains its structure after drying from the DMF mixture with a polymer solution, integrating the diamond particles into the polymer structure. The inset in Figure 5b shows the value of σ_0_ of the differential curves in the range of smaller q, normalized to DND content, vs. DND content in membranes. This value remains almost constant, with a slight tendency to decrease, meaning the additivity of SANS intensity from a diamond component is not quite universal. This slight decrease, similar to that described in [38], corresponds to an increase in the distribution uniformity of DND particles on a scale of 10–100 nm; therefore, there is a decrease in the nanosized diamond clusters fraction and a possible growth of submicron clusters.

The differential SANS curves in the range of smallest q were fitted by Guinier function (2)

**Figure 6:**
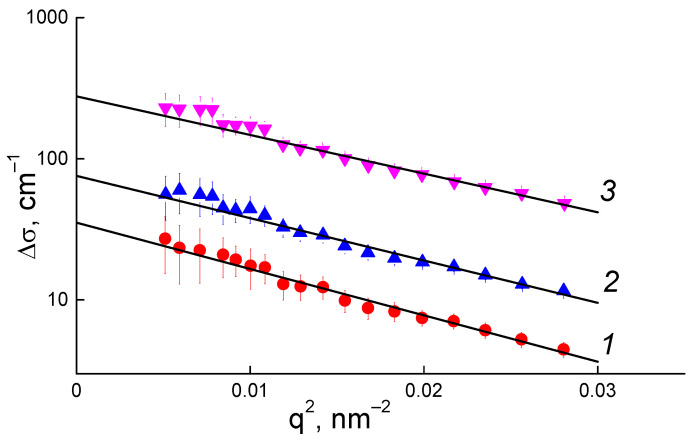
Guinier approximation of differential SANS curves for membranes with 0.25 wt.% (1); 1 wt.% (2) and 5.0 wt.% (3) of DND.

**Figure 7:**
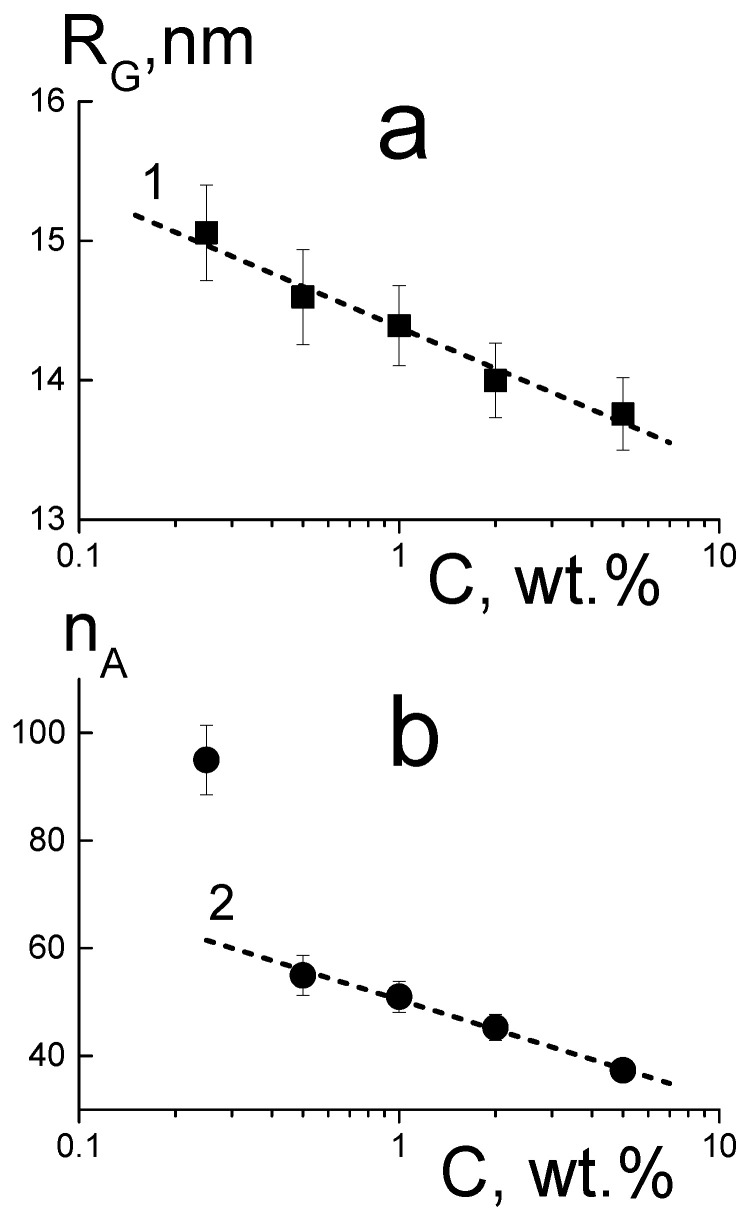
Gyration radii R_G_(C) (**a**) and aggregation numbers n_A_(C) (**b**) of diamond structures in membranes vs. DND content (C) from SANS data. Points are experimental data, dashed lines demonstrate logarithmic decrease for R_G_ (1) and n_A_ (2).

∆𝜎(q) = ∆𝜎_0_·exp(−(qR_G_)^2^/3), (2)

to find cross-sections at the low q approximation 𝛥𝜎_0_ and gyration radii R_G_ of diamond aggregates (Figure 6). The values of Δσ_0_ = (ΔK_DP_)^2^φv_P_n_A_ were then used to calculate the aggregation numbers of diamond particles n_A_ from the known contrast factor between diamonds and a polymer for neutrons (ΔK_DP_), volume fraction of diamonds (φ), and a volume of a single diamond particle (v_P_) with a diameter d_P_ = 4.5 nm. The parameters R_G_ and n_A_ were plotted and analyzed against the DND content (C) in membranes (Figure 7).

The parameters in Figure 7 are plotted in a logarithmic scale, where the logarithmic decrease of the aggregates’ sizes (Figure 7a) is detected according to (3):

R_G_(C) = R_G1_ − α_R_·ln(C), (3)

where the parameter R_G1_ = 14.38 ± 0.04 nm corresponds to the DND concentration C = C_1_ = 1 wt.%, and a coefficient α_R_ = 0.42 ± 0.04 characterizes the dynamics of the radius changing with the DND content.

The aggregation numbers (Figure 7b) also decrease logarithmically (4) with the exception of the membrane with the lowest DND content (0.25 wt.%):

n_A_(C) = n_A1_ − α_n_·ln(C), (4)

with the corresponding parameters n_A1_ = 50.4 ± 0.5, α_n_ = 8.0 ± 0.4.

Equations (3) and (4) demonstrate the correlation of the structural parameters with linear dependence (5):

n_A_(C) − n_A1_ = (α_n_/α_R_)[R_G_(C) − R_G1_)], (5)

where the ratio of the parameters α_n_/α_R_ = 19.0 ± 2.1 = dn_A_/dR_G_ is a derivative of the aggregation number with respect to the gyration radius.

Thus, the enrichment of membranes with diamonds, contrary to the expected increase in diamond aggregation, leads to a decrease in their gyration radius and the mass of nanosized aggregates. This fact demonstrates an increase in the integration of diamonds into the polymer, which is supported by the presence of sulfonic acid groups on the DND surface. So, sulfonated DNDs were found to be quite compatible with a short side chain PFSA ionomer. DNDs are well integrated into the polymer structure, saving the mechanical properties and improving ionic conductivity of compositional membranes.

### 3.5. Surface Structure of Membranes from Scanning Electron Microscopy

**Figure 8:**
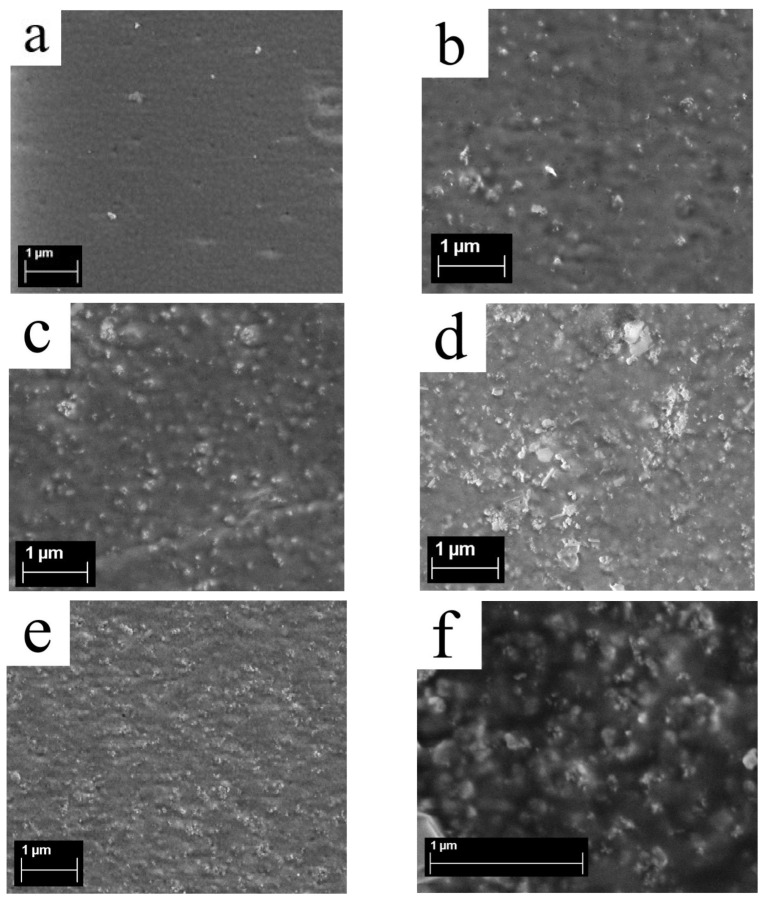
SEM images of Aquivion^®^-type compositional membranes with DND-S: (**a**) 0% DND (without nanodiamonds); (**b**) 0.5 wt.% DND; (**c**) 1 wt.% DND; (**d**) 2 wt.% DND; (**e**) and (**f**) 5 wt.% DND (different scales).

The prepared Aquivion^®^-type membranes with DND-S (0, 0.5, 1, 2, and 5 wt.% DND) were studied by SEM. A cellular structure with globule sizes of ~150 nm is detected on the surface of membrane without diamonds (Figure 8a), already reported [38], which is also consistent with earlier atomic force microscopy (AFM) data [17]. On the surface of the composite membranes, diamond clusters are found, with maximal sizes from ~100 to 200–300 nm, which rise as the content of DND-S in the membrane increases (Figure 8 b–f). Even in the presence of 5 wt.% of DND-S, diamonds on the membrane’s surface are in the form of rough clusters of up to 100–150 nm in size (Figure 8e,f), which is smaller than in the case of the initial DND-Z+ [38], and rather uniformly distributed over the membrane’s area. Large and dense clusters are not detected. Thus, a 10-fold rise of DND content in the compositional membranes results in a few growths of the size of the submicron DND clusters, leading mainly to a denser distribution of DND-S clusters, which confirms our assumption above, based on the SANS data. Additional SEM images for compositional Aquivion^®^-type membranes with DND-S are provided in Figures S2–S9.

Apparently, the nature of DND-S, having both negative and positive charges on its surface, provides the most homogeneous distribution among all types of DNDs used (DND Z+, DND Z−, and DND-S). This is also consistent with the proton conductivity data, where a prolonged maximum is observed without a significant drop, even at 5 wt.% of DND-S.

## 4. Conclusions

Sulfonated nanodiamonds (DND-S), prepared from DND Z+ (hydrogynated nanodiamonds) by partial sulfonation using oleum, integrate well into Aquivion^®^-type PFSA membranes. Compositional membranes with DND-S, prepared by a solution casting technique, demonstrate excellent mechanical properties and elevated proton conductivity. The optimal content of DND-S in compositional membranes is ~1 wt.%, where the maximum of proton conductivity at 50 °C is observed, supported with good rigidity and ultimate tensile strength. The maximum of proton conductivity value is shifted to larger DND content, compared to initial DND Z+, due to more homogeneous DND-S distribution in a polymer matrix. This is determined by negatively charged –SO_3_^−^—groups on the DND surface, while having a positive charge in total, and allows the introduction of more DND additive with ionogenic groups along with the preservation of the membranes’ structure.

Small-angle neuron scattering of compositional membranes with DND-S demonstrate a retaining of the fine structure of proton-conducting channels in membranes. DND-S particles form branched rough clusters in the polymer matrix, which aggregation numbers decrease from ~95 to ~40, while the DND content in membrane rises from 0.25 wt.% to 5 wt.%.

The effective introduction of DND-S into Aquivion^®^-type membranes allowed us to obtain membranes with improved properties for use in hydrogen fuel cells and opens a new field of further investigations.

## Data Availability

Not applicable.

## Supplementary Materials

The following supporting information can be downloaded at: https://www.mdpi.com/article/10.3390/membranes13080712/s1, Figure S1: TGA tests of SSC-1 (EW 790 g-eq/mol, blue) and SSC-2 (EW 870 g-eq/mol, green) membranes, Figure S2: SEM image of Aquivion^®^-type compositional membrane with 0.5 wt.% of DND-S, Figure S3: SEM image of Aquivion^®^-type compositional membrane with 0.5 wt.% of DND-S, Figure S4: SEM image of Aquivion^®^-type compositional membrane with 1 wt.% of DND-S, Figure S5: SEM image of Aquivion^®^-type compositional membrane with 1 wt.% of DND-S, Figure S6: SEM image of Aquivion^®^-type compositional membrane with 2 wt.% of DND-S, Figure S7: SEM image of Aquivion^®^-type compositional membrane with 2 wt.% of DND-S, Figure S8: SEM image of Aquivion^®^-type compositional membrane with 5 wt.% of DND-S, Figure S9: SEM image of Aquivion^®^-type compositional membrane with 5 wt.% of DND-S.

## References

[1] Filippov S.P., Yaroslavtsev A.B. (2021). Hydrogen energy: Development prospects and materials. Russ. Chem. Rev..

[2] Zhang F., Zhao P., Niu M., Maddy J. (2016). The survey of key technologies in hydrogen energy storage. Int. J. Hydrogen Energy.

[3] Kusoglu A., Weber A.Z. (2017). New insights into perfluorinated sulfonic-acid ionomers. Chem. Rev..

[4] Alent’ev A.Y., Volkov A.V., Vorotyntsev I.V., Maksimov A.L., Yaroslavtsev A.B. (2021). Membrane technologies for decarbonization. Membr. Membr. Technol..

[5] Sun C., Negro E., Vezzù K., Pagot G., Cavinato G., Nale A., Bang Y.H., Di Noto V. (2019). Hybrid inorganic-organic proton-conducting membranes based on SPEEK doped with WO3 nanoparticles for application in vanadium redox flow batteries. Electrochim. Acta.

[6] Fathabadi H. (2018). Utilizing solar and wind energy in plug-in hybrid electric vehicles. Energy Convers. Manag..

[7] Dunn B., Kamath H., Tarascon J.-M. (2011). Electrical Energy Storage for the Grid: A Battery of Choices. Science.

[8] Sun C., Zhang H. (2022). Review the development of first-generation redox flow batteries: Iron-chromium system. ChemSusChem.

[9] Ivanchev S.S., Myakin S.V. (2010). Polymer membranes for fuel cells: Manufacture, structure, modification, properties. Russ. Chem. Rev..

[10] Danilczuck M., Lancucki L., Schlick S., Hamrock S.J., Haugen G.M. (2012). In-depth profiling of degradationin processes in a fuel cell: 2D spectral-spatial FTIR spectra of Nafion membranes. ACS Macro Lett..

[11] Banergjee S., Curtin D.E. (2004). Nafion^®^ perfluorinated membranes in fuel cell. J. Fluor. Chem..

[12] Hiesgen R., Aleksandrova E., Meichsner G., Wehl I., Roduner E., Friedrich K.A. (2009). High-resolution imaging of ion conductivity of Nafion^®^ membranes with electrochemical atomic force microscopy. Electrochim. Acta.

[13] Xie T. (2010). Tunable polymer multi-shape memory effect. Nature.

[14] Komarov P.V., Khalatur P.G., Khokhlov A.R. (2013). Large-scale atomistic and quantum-mechanical simulations of a Nafion membrane: Morphology, proton solvation and charge transport. Beilstein J. Nanotechnol..

[15] Xiao P., Li J., Cho C. (2014). Experimental investigation and discussion on the mechanical endurance limit of Nafion membrane used in proton membrane fuel cell. Energies.

[16] Primachenko O.N., Marinenko E.A., Odinokov A.S., Kononova S.V., Kulvelis Y.V., Lebedev V.T. (2021). State of the art and prospects in the development of proton conducting perfluorinated membranes with short side chains: A review. Polym. Adv. Technol..

[17] Kulvelis Y.V., Ivanchev S.S., Lebedev V.T., Primachenko O.N., Likhomanov V.S., Török. G. (2015). Structure characterization of perfluorosulfonic short side chain polymer membranes. RSC Adv..

[18] Primachenko O.N., Odinokov A.S., Barabanov V.G., Tyul’mankov V.P., Marinenko E.A., Gofman I.V., Ivanchev S.S. (2018). Relationship between the morphology, nanostructure, and strength properties of Aquivion^®^ type perfluorinated proton-conducting membranes prepared by casting from solution. Russ. J. Appl. Chem..

[19] Safronova E.Y., Osipov A.K., Yaroslavtsev A.B. (2018). Short Side Chain Aquivion Perfluorinated Sulfonated Proton-Conductive Membranes: Transport and Mechanical Properties. Pet. Chem..

[20] Xu F., Mu S. (2014). Nanoceramic Oxide Hybrid Electrolyte Membranes for Proton Exchange Membrane Fuel Cells. J. Nanosci. Nanotechnol..

[21] Wong C.Y., Wong W.Y., Ramya K., Khali M., Loh K.S., Daud K.L., Lim W.R.W., Walvekar R., Kadhum A.A.H. (2019). Additives in proton exchange membranes for low and high-temperature fuel cell applications: A review. Int. J. Hydrogen Energy.

[22] Bakangura E., Wu L., Ge L., Yang Z., Xu T. (2016). Mixed matrix proton exchange membranes for fuel cells: State of the art and perspectives. Prog. Polym. Sci..

[23] Zhang H., Hu Q., Zheng X., Yin Y., Wu H., Jiang Z. (2019). Incorporating phosphoric acid-functionalized polydopamine into Nafion polymer by in situ sol-gel method for enhanced proton conductivity. J. Membr. Sci..

[24] Ying Y.P., Kamarudin S.K., Masdar M.S. (2018). Silica-related membranes in fuel cell applications: An overview. Int. J. Hydrogen Energy.

[25] Park J.-S., Shin M.-S., Kim C.-S. (2017). Proton exchange membranes for fuel cell operation at low relative humidity and intermediate temperature: An updated review. Curr. Opin. Electrochem..

[26] Pethaiah S.S., Kalaignan G.P., Ulaganathan M., Arunkumar J. (2011). Preparation of durable nanocatalyzed MEA for PEM fuel cell applications. Ionics.

[27] Kim D.J., Jo M.J., Nam S.Y. (2015). A review of polymer-nanocomposite electrolyte membranes for fuel cell application. J. Ind. Eng. Chem..

[28] Porozhnyy M.V., Shkirskaya S.A., Butylskii D.Y., Dotsenko V.V., Safronova E.Y., Yaroslavtsev A.B., Deabate S., Huguet P., Nikonenko V.V. (2021). Physicochemical and electrochemical characterization of Nafion-type membranes with embedded silica nanoparticles: Effect of functionalization. Electrochim. Acta.

[29] Yadav R., Subhash A., Chemmenchery N., Kandasubramanian B. (2018). Graphene and Graphene Oxide for Fuel Cell Technology. Ind. Eng. Chem. Res..

[30] Nunn N., Torelli M., McGuire G., Shenderova O. (2017). Nanodiamond: A high impact nanomaterial. Curr. Opin. Solid State Mater. Sci..

[31] Yang N., Foord J.S., Jiang X. (2016). Diamond electrochemistry at the nanoscale: A review. Carbon.

[32] Postnov V.N., Mel’nikova N.A., Shul’meister G.A., Novikov A.G., Murin I.V., Zhukov A.N. (2017). Nafion- and Aquivion-Based Nanocomposites Containing Detonation Nanodiamonds. Russ. J. Gen. Chem..

[33] Aleksenskiy A.E., Eydelman E.D., Vul’ A.Y. (2011). Deagglomeration of detonation nanodiamonds. Nanosci. Nanotechnol. Lett..

[34] Kulvelis Y.V., Primachenko O.N., Odinokov A.S., Shvidchenko A.V., Bairamukov V.Y., Gofman I.V., Lebedev V.T., Ivanchev S.S., Vul A.Y., Kuklin A.I. (2020). Composite proton-conducting membranes with nanodiamonds. Fuller. Nanotub. Carbon Nanostruct..

[35] Primachenko O.N., Kulvelis Y.V., Lebedev V.T., Odinokov A.S., Bayramukov V.Y., Marinenko E.A., Gofman I.V., Shvidchenko A.V., Vul A.Y., Ivanchev S.S. (2020). Perfluorinated proton-conducting membrane composites with functionalized nanodiamonds. Membr. Membr. Technol..

[36] Kulvelis Y.V., Primachenko O.N., Gofman I.V., Odinokov A.S., Shvidchenko A.V., Yudina E.B., Marinenko E.A., Lebedev V.T., Vul A.Y. (2021). Modification of the mechanism of proton conductivity of the perfluorinated membrane copolymer by nanodiamonds. Russ. Chem. Bull. Int. Ed..

[37] Williams O.A., Hees J., Dieker C., Jager W., Kirste L., Nebel C.E. (2010). Size-Dependent Reactivity of Diamond Nanoparticles. ACS Nano.

[38] Primachenko O.N., Kulvelis Y.V., Odinokov A.S., Glebova N.V., Krasnova A.O., Antokolskiy L.A., Nechitailov A.A., Shvidchenko A.V., Gofman I.V., Marinenko E.A. (2022). New Generation of Compositional Aquivion^®^-Type Membranes with Nanodiamonds for Hydrogen Fuel Cells: Design and Performance. Membranes.

[39] Simari C., Stallworth P., Peng J., Coppola L., Greenbaum S., Nicotera I. (2019). Graphene oxide and sulfonated-derivative: Proton transport properties and electrochemical behavior of Nafion-based nanocomposites. Electrochim. Acta.

[40] Sgambetterra M., Brutti S., Allodi V., Mariotto G., Panero S., Navarra M.A. (2016). Critical filler concentration in sulfated titania-added Nafion™ membranes for fuel cell applications. Energies.

[41] Vinothkannan M., Kim A.R., Ramakrishnan S., Yu Y.-T., Yoo D.J. (2021). Advanced Nafion nanocomposite membrane embedded with unzipped and functionalized graphite nanofibers for high-temperature hydrogen-air fuel cell system: The impact of filler on power density, chemical durability and hydrogen permeability of membrane. Composites Part B..

[42] Rambabu G., Nagaraju N., Bhat S.D. (2016). Functionalized fullerene embedded in Nafion matrix: A modified composite membrane electrolyte for direct methanol fuel cells. Chem. Eng. J..

[43] Kuznetsov O., Sun Y., Thaner R., Bratt A., Shenoy V., Wong M.S., Jones J., Billups W.E. (2012). Water-soluble nanodiamond. Langmuir.

[44] Lei Y., Huang Q., Gan D., Huang H., Chen J., Deng F., Liu M., Li X., Zhang X., Wei Y. (2020). A novel one-step method for preparation of sulfonate functionalized nanodiamonds and their utilization for ultrafast removal of organic dyes with high efficiency: Kinetic and isotherm studies. J. Environ. Chem. Eng..

[45] Petit T., Girard H.A., Trouvé A., Batonneau-Gener I., Bergonzo P., Arnault J.-C. (2013). Surface transfer doping can mediate both colloidal stability and selfassembly of nanodiamonds. Nanoscale.

[46] Ginés L., Mandal S., Cheng C.-L., Sow M., Williams O.A. (2017). Positive zeta potential of nanodiamonds. Nanoscale.

[47] Vul A.Y., Eidelman E.D., Aleksenskiy A.E., Shvidchenko A.V., Dideikin A.T., Yuferev V.S., Lebedev V.T., Kul’velis Y.V., Avdeev M.V. (2017). Transition sol-gel in nanodiamond hydrosols. Carbon.

[48] Primachenko O.N., Odinokov A.S., Marinenko E.A., Kulvelis Y.V., Barabanov V.G., Kononova S.V. (2021). Influence of sulfonyl fluoride monomers on the mechanism of emulsion copolymerization with the preparation of proton-conducting membrane precursors. J. Fluor. Chem..

[49] Delgado A.V., González-Caballero F., Hunter R.J., Koopal L.K., Lyklema J. (2007). Measurement and interpretation of electrokinetic phenomena. J. Coll. Interf. Sci..

[50] Kuklin A.I., Ivankov O.I., Rogachev A.V., Soloviov D.V., Islamov A.K., Skoi V.V., Kovalev Y.S., Vlasov A.V., Ryzhykau Y.L., Soloviev A.G. (2021). Small-Angle Neutron Scattering at the Pulsed Reactor IBR-2: Current Status and Prospects. Crystallogr. Rep..

[51] Kuklin A.I., Ivankov A.I., Soloviov D.V., Rogachev A.V., Kovalev Y.S., Soloviev A.G., Islamov A.K., Balasoiu M., Vlasov A.V., Kutuzov S.A. (2018). High-throughput SANS experiment on two-detector system of YuMO spectrometer. J. Phys. Conf. Ser..

[52] Soloviev A.G., Solovjeva T.M., Ivankov O.I., Soloviov D.V., Rogachev A.V., Kuklin A.I. (2017). SAS program for two-detector system: Seamless curve from both detectors. J. Phys. Conf. Ser..

[53] Petit T., Puskar L. (2018). FTIR spectroscopy of nanodiamonds: Methods and interpretation. Diam. Rel. Mat..

[54] Danilczuk M., Lin L., Schlick S., Hamrock S.J., Schaberg M.S. (2011). Understanding the fingerprint region in the infra-red spectra of perfluorinated ionomer membranes and corresponding model compounds: Experiments and theoretical calculations. J. Pow. Sourc..

[55] Kulvelis Y.V., Ivanchev S.S., Primachenko O.N., Lebedev V.T., Marinenko E.A., Ivanova I.N., Kuklin A.I., Ivankov O.I., Soloviov D.V. (2016). Structure and property optimization of perfluorinated short side chain membranes for hydrogen fuel cells using orientational stretching. RSC Adv..

[56] Teixeira J. (1988). Small-angle scattering by fractal systems. J. Appl. Cryst..

[57] Grigoriev S.V., Iashina E.G., Bairamukov V.Y., Pipich V., Radulescu A., Filatov M.V., Pantina R.A., Varfolomeeva E.Y. (2020). Switch of fractal properties of DNA in chicken erythrocytes nuclei by mechanical stress. Phys. Rev. E.

[58] Iashina E.G., Varfolomeeva E.Y., Pantina R.A., Bairamukov V.Y., Kovalev R.A., Fedorova N.D., Pipich V., Radulescu A., Grigoriev S.V. (2021). Bifractal structure of chromatin in rat lymphocyte nuclei. Phys. Rev. E.

[59] Lebedev V., Kulvelis Y., Kuklin A., Vul A. (2016). Neutron Study of Multilevel Structures of Diamond Gels. Cond. Matter..

[60] Avdeev M.V., Tomchuk O.V., Ivankov O.I., Alexenskii A.E., Dideikin A.T., Vul A.Y. (2016). On the structure of concentrated detonation nanodiamond hydrosols with a positive ζ potential: Analysis of small-angle neutron scattering. Chem. Phys. Lett..

[61] Tomchuk O.V., Mchedlov-Petrossyan N.O., Kyzyma O.A., Kriklya N.N., Bulavin L.A., Zabulonov Y.L., Ivankov O.I., Garamus V.M., Ōsawa E., Avdeev M.V. (2022). Cluster-cluster interaction in nanodiamond hydrosols by small-angle scattering. J. Mol. Liq..

