# Supplementary material for: Improving PFSA Membranes Using Sulfonated Nanodiamonds
Source: Membranes (Basel). 2023 Aug 1;13(8):712. doi: 10.3390/membranes13080712 (PMC10456736; doi:10.3390/membranes13080712)
Supplement: Supplementary file 1 [file membranes-13-00712-s001.zip › membranes-2470258-supplementary.pdf]

Supplementary information

Improving PFSA membranes using sulfonated nanodiamonds

Alexandr V. Shvidchenko [1,*], Alexei S. Odinokov [2], Oleg N. Primachenko [3], Iosif V. Gofman [3], Natalia P. Yevlampieva [4], Elena A. Marinenko [3], Vasily T. Lebedev [5], Alexander I. Kuklin [6] and Yuri V. Kulvelis [5,*]

[1] Ioffe Institute, 194021 St. Petersburg, Russia
[2] Russian Research Center of Applied Chemistry, 193232 St. Petersburg, Russia
[3] Institute of Macromolecular Compounds, Russian Academy of Sciences, 199004 St. Petersburg, Russia
[4] Physical Faculty, St. Petersburg State University, 198504 St. Petersburg, Russia
[5] Petersburg Nuclear Physics Institute named by B.P. Konstantinov of National Research Center "Kurchatov Institute", 188300 Gatchina, Russia
[6] Frank Laboratory of Neutron Physics, Joint Institute for Nuclear Research, 141980 Dubna, Russia
* Correspondence: avshvid@mail.ioffe.ru, kulvelis_yv@pnpi.nrcki.ru

Thermogravimetric analysis (TGA)

We have performed TGA tests of some Aquivion®-type membranes without diamonds: SSC-1 membrane with EW 790 g-eq/mol and SSC-2 membrane with EW 870 g-eq/mol. TGA was carried out in the temperature range 50-1000 °C for membranes SSC-1 and SSC-2 with different chemical composition (equivalent weight, EW). Thermal stability and the process of desulfonation were studied. Figure S1 shows the results of thermal-oxidative degradation of membrane copolymers in air.

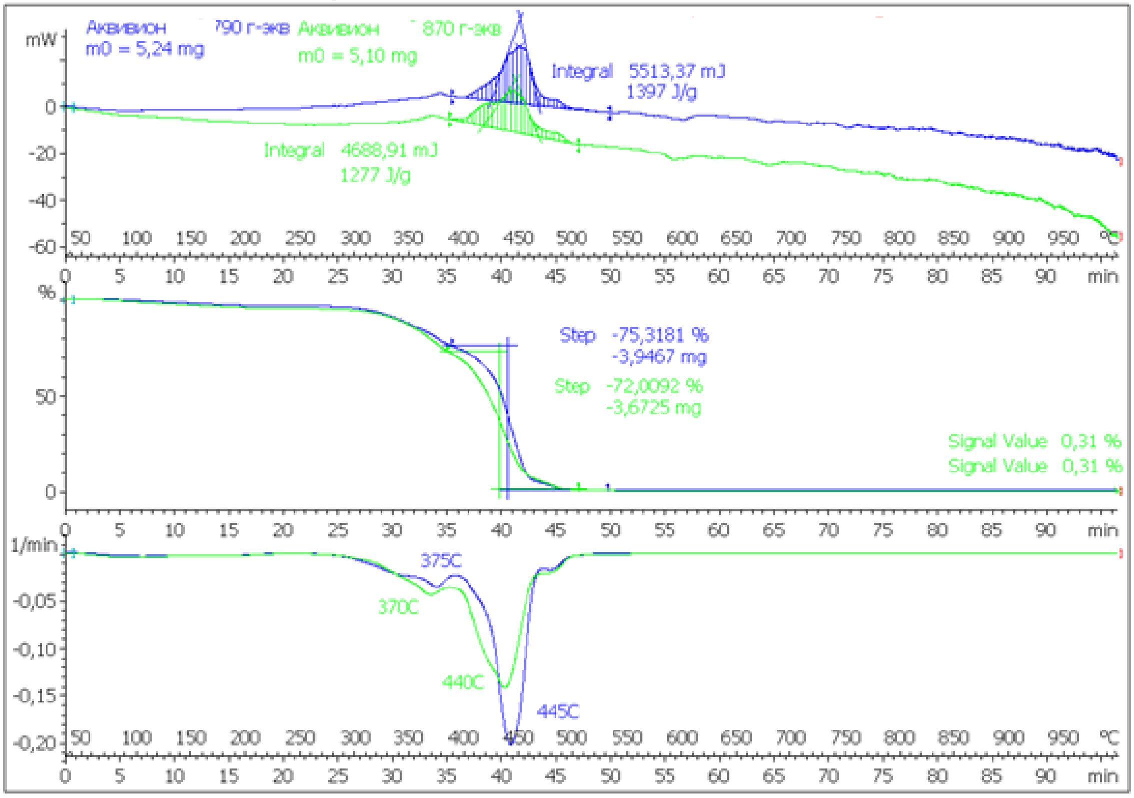

Figure S1. TGA tests of SSC-1 (EW 790 g-eq/mol, blue) and SSC-2 (EW 870 g-eq/mol, green) membranes.

3 main stages of membranes' destruction were identified. The total weight loss of all membranes up to 300 °C is associated with water evaporation. The weight loss at 300–400 °C is associated with desulfurization and decomposition of the side chains, and the weight loss above 400 °C is due to thermal destruction of the backbone of the copolymer.

Scanning electron microscopy (SEM)

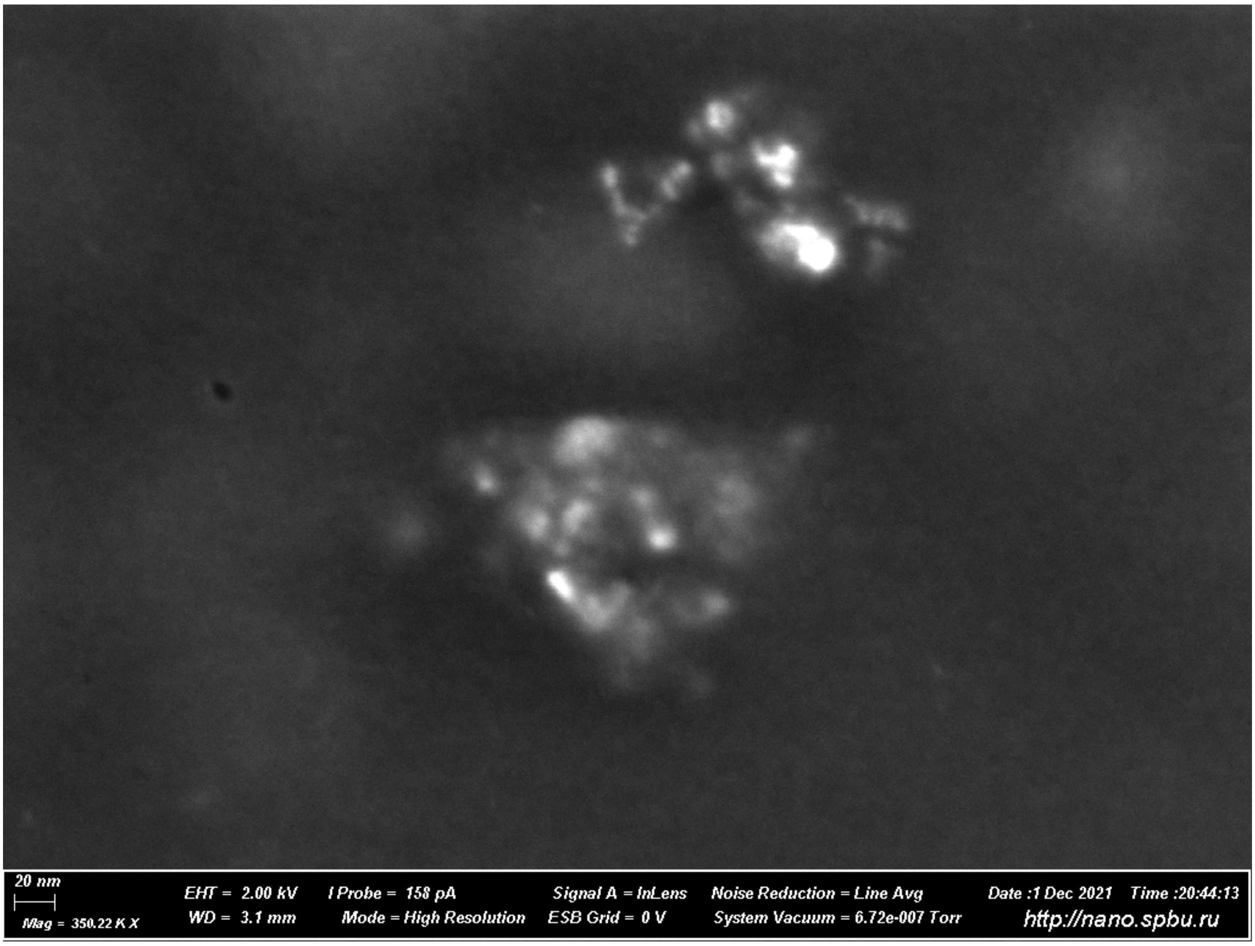

Figure S2. SEM image of Aquivion®-type compositional membrane with 0.5 wt.% of DND-S.

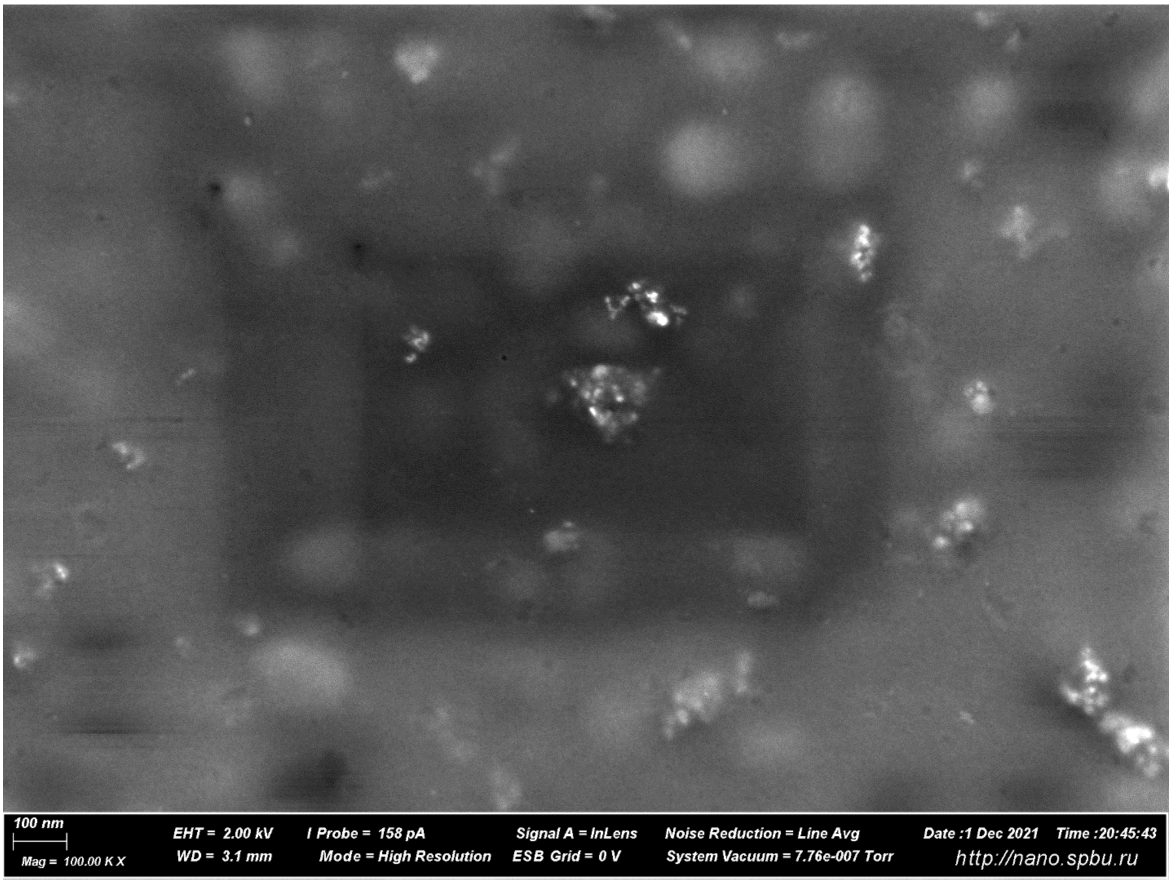

Figure S3. SEM image of Aquivion®-type compositional membrane with 0.5 wt.% of DND-S.

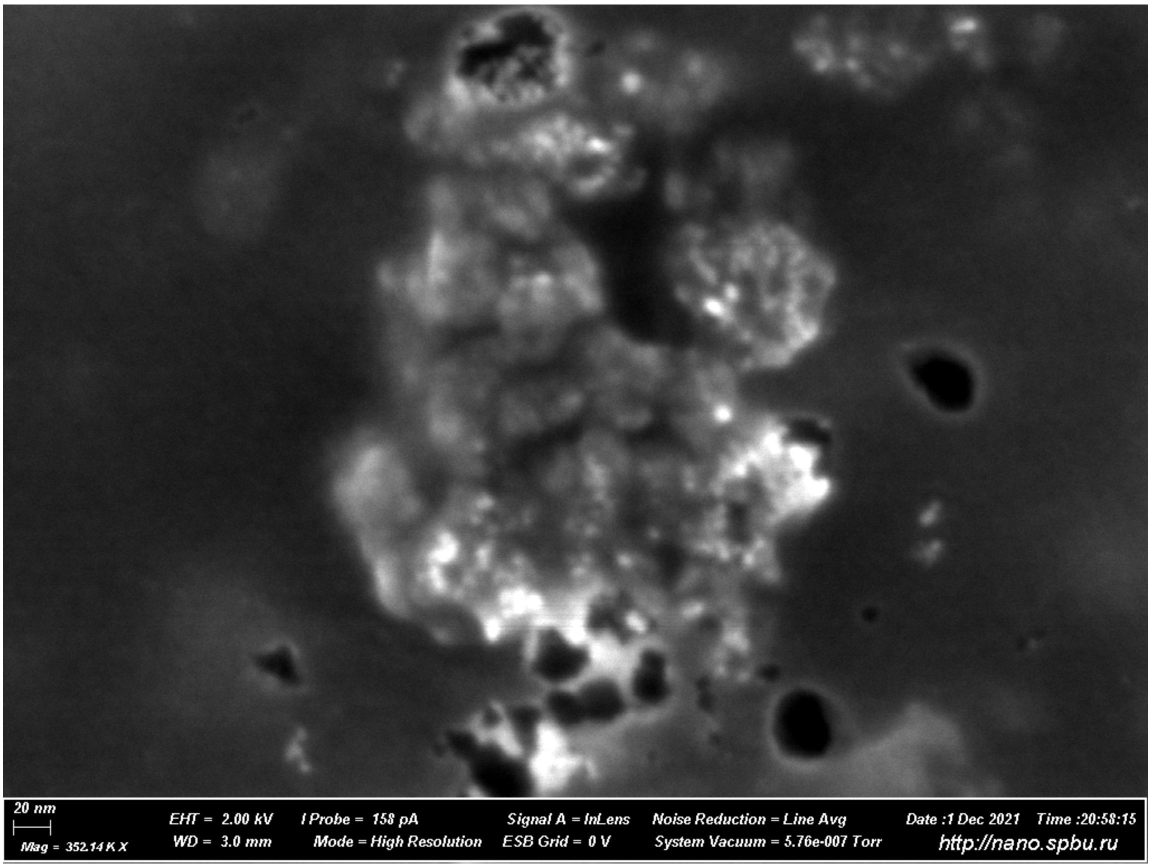

Figure S4. SEM image of Aquivion®-type compositional membrane with 1 wt.% of DND-S.

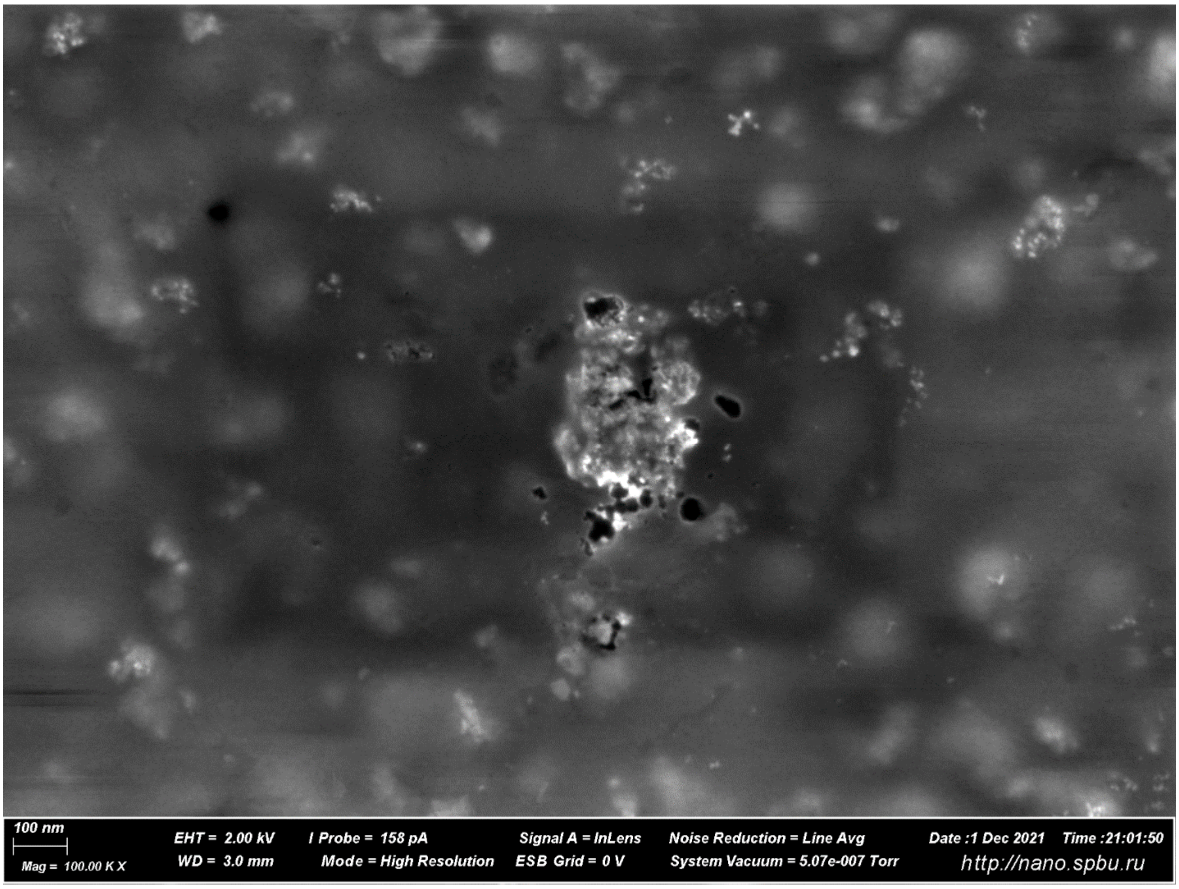

Figure S5. SEM image of Aquivion®-type compositional membrane with 1 wt.% of DND-S.

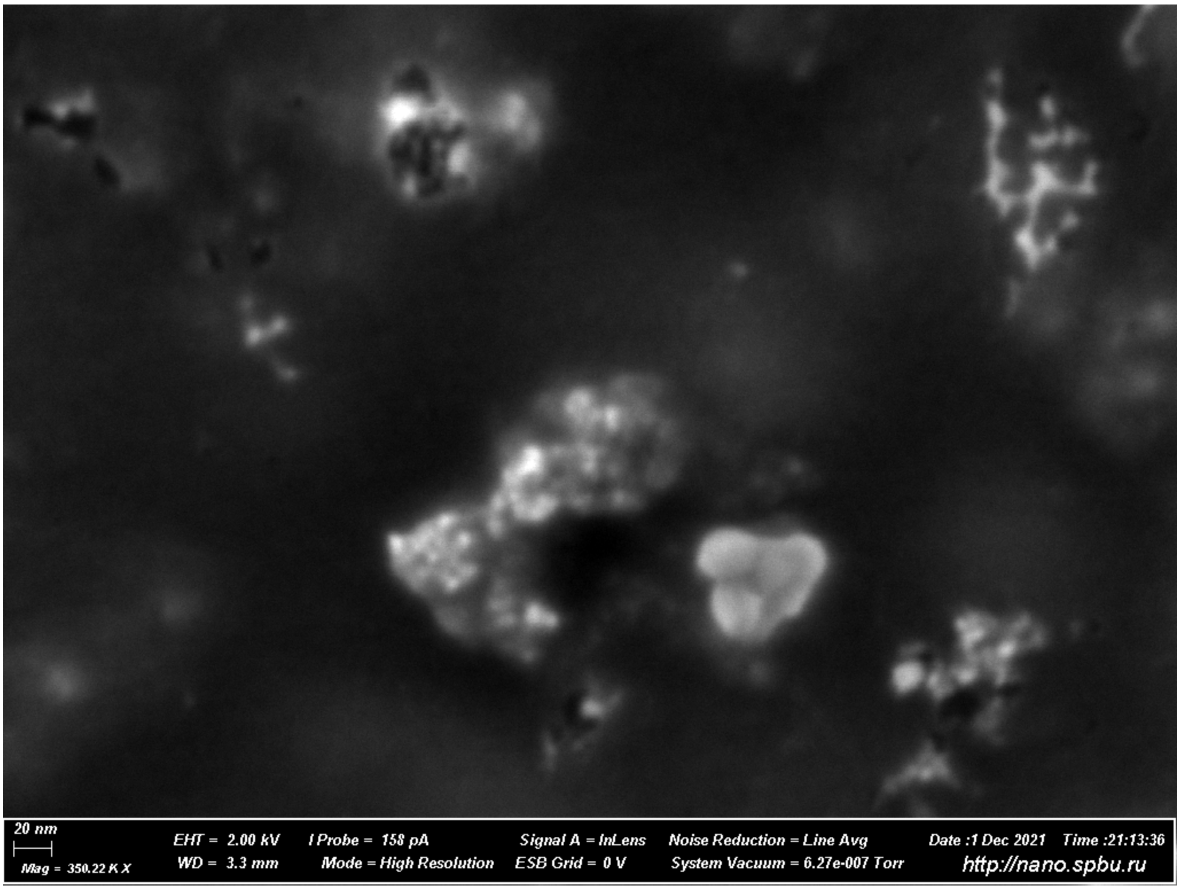

Figure S6. SEM image of Aquivion®-type compositional membrane with 2 wt.% of DND-S.

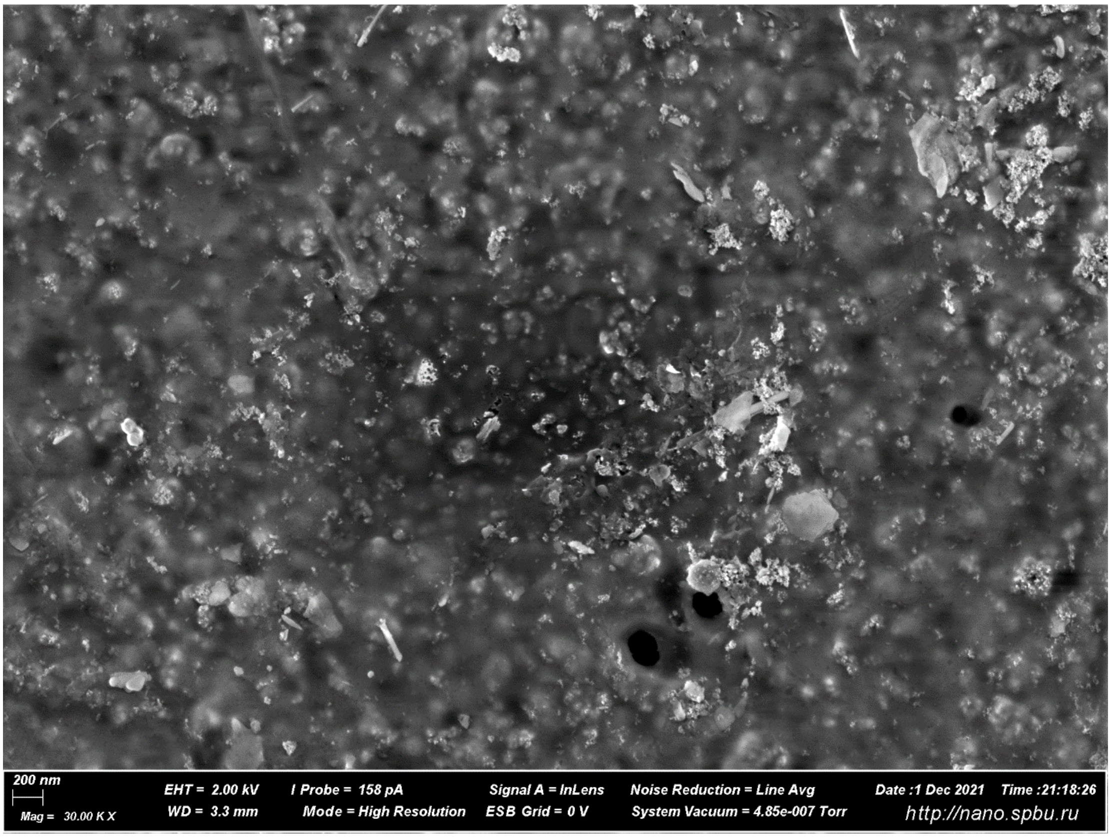

Figure S7. SEM image of Aquivion®-type compositional membrane with 2 wt.% of DND-S.

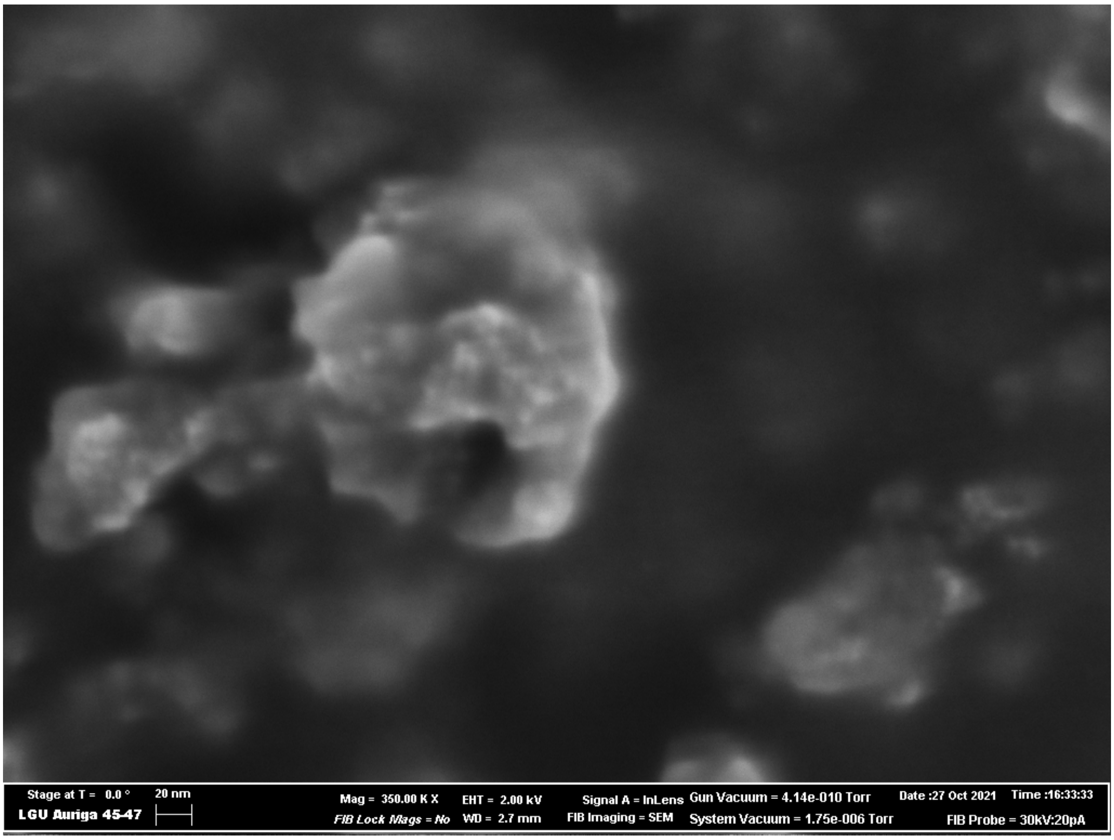

Figure S8. SEM image of Aquivion®-type compositional membrane with 5 wt.% of DND-S.

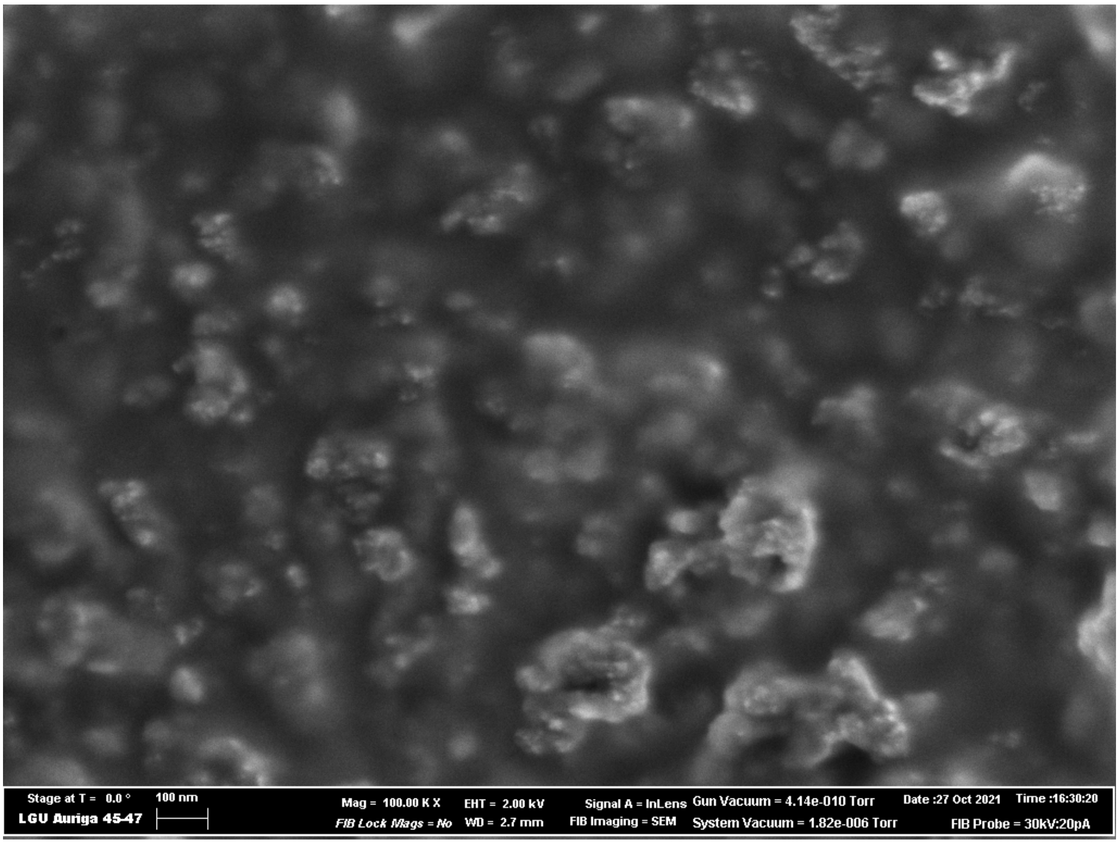

Figure S9. SEM image of Aquivion®-type compositional membrane with 5 wt.% of DND-S.
